# Biomarkers of Joint Damage in Osteoarthritis: Current Status and Future Directions

**DOI:** 10.1155/2021/5574582

**Published:** 2021-03-09

**Authors:** Rajkamal Kumavat, Vijay Kumar, Rajesh Malhotra, Hemant Pandit, Elena Jones, Frederique Ponchel, Sagarika Biswas

**Affiliations:** ^1^Department of Integrative and Functional Biology, CSIR-Institute of Genomics & Integrative Biology, Mall Road, -110007, Delhi, India; ^2^Academy of Scientific and Innovative Research (AcSIR), Ghaziabad 201002, India; ^3^All India Institute of Medical Sciences, Ansari Nagar, New Delhi 110029, India; ^4^Leeds Institute of Rheumatic and Musculoskeletal Medicine, The University of Leeds, Leeds, UK

## Abstract

Osteoarthritis (OA) is a disease of the whole joint organ, characterized by the loss of cartilage, and structural changes in bone including the formation of osteophytes, causing disability and loss of function. It is also associated with systemic mediators and low-grade inflammation. Currently, there is negligible/no availability of specific biomarkers that can be used to facilitate the diagnosis and treatment of OA. The most unmet clinical need is, however, related to the monitoring of disease progression over a short period that can be used in clinical trials. In this review, the value of biomarkers identified over the past decade has been highlighted. These biomarkers are associated with the synthesis and breakdown of cartilage, including collagenous and noncollagenous biomarkers, inflammatory and anti-inflammatory biomarkers, expressed in the biological fluid such as serum, synovial fluid, and urine. Broad validation of novel and clinically applicable biomarkers and their involvement in the pathways are particularly needed for early-stage diagnosis, monitoring disease progression, and severity and examining new drugs to mitigate the effects of this highly prevalent and debilitating condition.

## 1. Introduction

Osteoarthritis (OA) is a group of pathologies involving joints deformities, degeneration of articular cartilage, subchondral sclerosis, osteophytes formation, and joint structural deterioration that causes disability and joint pain [1]. In 2016, Osteoarthritis Research Society International (OARSI) submitted a white paper supporting the argument that OA is a serious disease because it affects the quality of life associated with increased risk of mortality and affects an economical burden to society [2]. With an estimated 303 million people affected worldwide, most people over the age of 60 have evidence of OA but it is estimated that 80% of the population has radiographic evidence, and symptomatic OA occurs in only 25% of people. According to the Global Burden of Disease (GBD) 2017 studies, the prevalence of knee OA is positively correlated with increased age, and radiographic knee OA is more prevalent compared to systematic knee OA [3]. The population-based studies have shown that the global prevalence of knee OA was 16.0% in individuals over 15 years of age and 22.9% in individuals over 40 years of age [4]. According to a National Health Interview Survey, approximately 14 million people are affected by systematic knee OA in the United States [5]. The prevalence rate of OA in India was found to be around 22% to 36%, and the prevalence of OA in the female gender is 31.6%, with the associate factors for OA that are obesity (*p* = 0.04), age (*p* = 0.001), and sedentary work (*p* = 0.0001) [6]. Epidemiologic studies have highlighted responsible risk factors, systemic factors (obesity, gender, genetic predisposition, etc.), and joint-related risk factors (joint injuries, joint misalignment) associated with OA development and progress [7]. OA is usually diagnosed by clinical manifestations (pain, swelling, morning stiffness for <30 minutes), X-rays, and magnetic resonance imaging (MRI). More recent advances in understanding OA have stemmed from epidemiologic studies using MRI, highlighting a very high frequency of pathology of cartilage, menisci, subchondral bone, and synovium [8]. Importantly, these studies have established the relevance of these tissues to joint pain associations, with pathology ranging from minor tissue lesions to severe total joint deviations. MRI provides additional information in the complex condition but is not used in the early-stage detection of OA. However, it is not available routinely and is usually restricted to clinical trials, where highly sensitive measures are needed to assess changes [9].

OA is a multifactorial disease where affected tissues undergo metabolic, structural, biochemical, and functional changes [10]. It is a result of the failure of chondrocytes to maintain homeostasis between synthesis and breakdown of the extracellular component such as proteoglycans and collagens, leading to inflammation of the synovium and joint capsule [11]. It is still unclear that which factors and processes initiate an imbalance between synthesis and breakdown of these components. These cartilage breakdown products are released into the synovial space, and the identity (type II collagen marker, COMP, etc)) of these products has been investigated as a potential biomarker for OA development [12].

## 2. The Clinical Need for Biomarkers in OA

**Figure 1:**
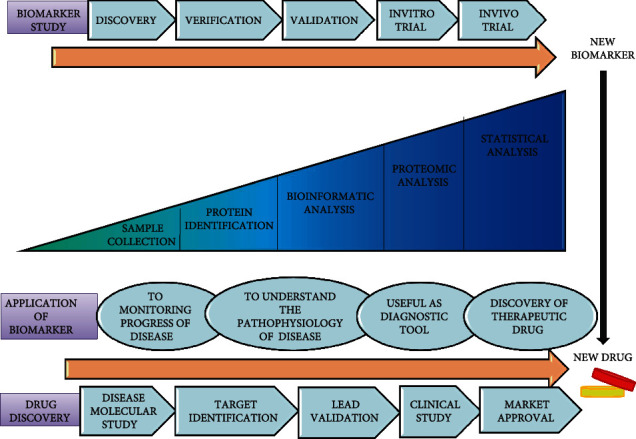
Biomarkers in drug discovery and development by using the proteomic approach.

The development of early OA interventions that could truly change the natural progression of OA is hampered by the lack of means to recognize early OA. It is being such a slow-progressing disease, and pathology is usually well established before symptoms are detected. There are no/negligible disease-modifying OA treatments to limit structural deterioration or clinical improvements in the disease, although recent advances may provide a real change in the future [13]. Also, another major clinical need is to identify biomarkers as shown in Figure 1 that may allow for monitoring disease progression in a shorter period, allowing for a more feasible clinical trial. To understand OA pathology involving molecular mechanisms, there is a need for specific molecules and pathways for early-stage detection and progression of OA. The breakdown products of cartilage and various cytokines that increase/decrease in the inflammation site as well as in circulating blood have been identified to be used as biomarkers for understating OA pathology [14], but the exact mechanism is still unknown. The discovery of new therapeutic drugs requires knowledge about the molecules and the associated pathways to understand the specific target bond. These biomarker molecules and related signaling pathways play role in regulating the maintenance of mature chondrocytes and turnover of articular cartilage, synovial inflammation, etc. [15].

## 3. Biomarker Candidates in OA

**Figure 2:**
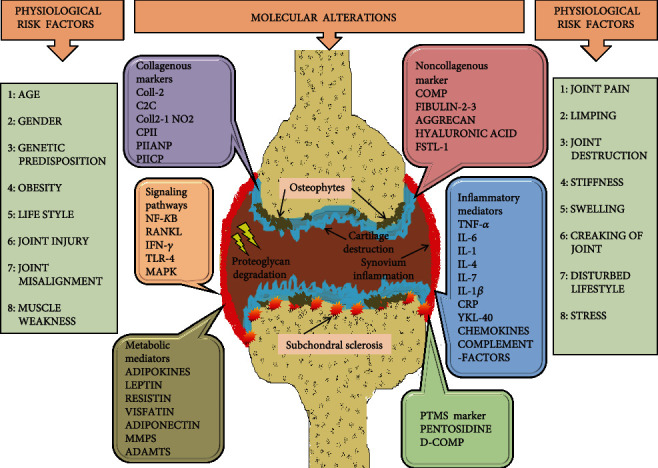
Physiological, molecular, and metabolic alterations are responsible for the cause of osteoarthritis. CPII: type II collagen propeptide; C2C: neoepitope of type II collagen; Coll 2-1: 9-amino acid peptide of type II collagen (nitrated form Coll 2-1 NO_2_); CTX: C-terminal telopeptide of collagen; PIIANP: N-propeptide IIA of type II collagen; PIICP: C-propeptide of collagen type II; COMP: cartilage oligomeric matrix protein; FSTL-1: follistatin-like protein 1; CRP: C-reactive protein; IL: interleukin; TNF: tumor necrosis factor; YKL-40: chitinase-3-like protein 1; ADMTs: A Disintegrin and Metalloproteinase with Thrombospondin motifs; MMP: matrix metalloproteinase; PTMs: posttranslational modifications; D-COMP: deaminated epitope of cartilage oligomeric matrix protein; NF-*κ*B: nuclear factor kappa light chain enhancer of activated B cells; RANKL: receptor activator of NF-*κ*B ligand; IFN-*γ*: interferon gamma; TLR4: toll-like receptor 4; MAPK: mitogen-activated protein kinase.

In 2001, the Biomarkers Definitions Working Group (BDWG) defined the biomarker as a “characteristic that is objectively measured and evaluated as an indicator of normal biological processes or pharmacological responses to a therapeutic intervention” [16]. The biomarker for OA should reflect dynamic and quantitative changes in joint tissues. There is accumulating evidence that these biomarkers can not only distinguish the cartilage breakdown changes in the cartilage of knee OA but can also diagnose functional, structural, and biochemical changes and associated damage in the bone and the subchondral tissue [17]. Several biomarkers have been proposed in OA over the years, for early-stage diagnosis, monitoring disease progression or severity, and for examining drug potential's efficacy. The identified biomarkers should be compatible with pain score, clinical, and radiological findings of disease, and the smallest changes in concentration of biomarkers should be related to disease severity and pathology [18]. To understand the role of biomarkers in clinical trials, it is necessary to standardize measurement methods such as sample collection time, sample storage status, physical activity, age, sex, disease progression, and information about medications which is used for the treatment of the patient [19]. For the discovery of drug development methods and meaningful use of biomarker in clinical trial and availability in the public, a guideline was issued by the Food and Drug Administration (FDA) that involved a pathway for identifying a biomarker with clinical outcome including in vitro and in vivo studies from an optimal dose of therapeutics, monitoring therapeutic responses, side effects, clinical validity (clear difference in both group), and clinical utility (effect on health) [20]. These biomarkers include proteins, metabolites, carbohydrate biomarkers, genomic biomarkers, cellular biomarkers, and imaging biomarkers and are usually measured in a selected body fluid such as blood, serum, urine, synovial fluid, and cartilage tissue. These markers are reflected in bone loss, chondrocytes erosion, and inflammation of joint tissue as shown in Figure 2 [21]. Several biochemical markers are usually the byproduct of cartilage breakdown, bone metabolism, lipid metabolism, and the component of extracellular matrix molecules which are released into the blood or body fluids during the process of tissue turnover. For this reason, different combinations of markers were reported to play an important role in the prognosis of the disease [22]. However, very few biomarkers (type II procollagen carboxy type 2-propeptide (CPII), N-propeptide type IIA collagen (PIIANP), collagen (uCTX-II), COLL2-1, and its nitrate form COLL2-1NO2 Fibulin-3 epitope, cartilage oligomeric matrix protein (COMP), pentosidine and adipokines, etc.) have been identified for prediction of the development of OA [23]. For easy access to these biomarkers, we have divided these biomarkers based on their tissue origin and metabolism and discussed each of these categories further as shown in Table 1. These are (a) collagenous biomarkers, (b) noncollagenous biomarkers, (c) inflammatory markers, (d) posttranslation modification biomarkers (e), and other biomarkers such as adipokines [23]. Based on previous studies and reports, we focused on how these biomarkers play role in OA progression and development involving multiple pathways, such as wnt signaling, NF-*κ*B pathway, and TLR-pathway [24].

### 3.1. Collagenous Biomarkers

Collagens are a large family of proteins found in the various types of connective tissues such as cartilage, bones, tendons, and muscles. During OA, breakdown and synthesis products of collagens have been evaluated as biomarkers (type2 collagen, nitrate form of Coll-2, procollagen–IIA, and urinary c-terminal crosslinking telopeptide (uCTX-II)) for the prediction of the development and progression of OA [25].

Type II collagen (Coll-II) is the major collagen of the cartilage, and its cleavage products (Coll2-1 and Coll2-1NO2) were upregulated by ~2-fold in the early stage of OA and were decreased in severe cases [26]. Coll2-1 is a peptide molecule released from type-II collagen during cartilage erosion and is a native form while Coll2-1NO2 is the nitrate form of Coll2-1. Peptide nitration is formed by the reaction of aromatic amino acid with peroxynitrite (ONOO-); peroxynitrite is a strong oxidant formed by the reaction of nitric oxide (NO) and superoxide (O_2_^−^). Both NO and (O_2_^−^) are formed in OA from macrophage cells and chondrocytes. Tyrosine, located in the alpha chain of type-II collagen, bind with nitrate, and form Coll2-1NO2 [27]. The nitrated form of Coll 2-1 reflects the oxidative-related cartilage degradation and inflammation in OA. The concentration of Coll2-1 in the serum remains constant throughout life but Coll2-1 and Coll2-1NO2 levels were elevated in the serum and synovial fluid (SF) of OA patients and lead cartilage degradation by increase oxidative stress in disease conditions. Coll2-1NO2 can be a useful tool to identify oxidative-related cartilage degradation, hence used for monitoring the effect of anti-inflammatory and antioxidant drugs on the cartilage [28]. Destruction of collagen type II and related pathology phenomenon is activated by the upregulation of WNT5A (wnt signaling pathway) in OA-chondrocytes. In clinical studies, it was found that silencing of WNT5A mRNA by small interfering RNA (siRNA) prevents degradation of Coll-2 [29]. In another study, treatment of OA by viscosupplementation with hyaluronic acid, the Coll2-1 level was observed to be reduced in the serum of OA, resulting in the prevention of collagen destruction in OA, suggesting that Coll2-1 as a predictive marker for diagnosis of OA [30].

Procollagen-IIA is an N-terminal propeptide, cleaved by procollagen-N and procollagen-C proteinases, producing procollagen type-N-propeptide (PIIANP) and procollagen-type-C propeptide (PIICP) [31]. PIIANP levels were downregulated in serum and OASF compared to healthy control. Lower PIIANP was associated with greater osteophytes and joint space contraction values, indicating that systemic PIIANP does not reflect the local collagen tissue in knee OA, but rather there is a more global cartilage anabolic reaction. Proanabolic agents PIIANP may be suitable for treatment therapy to prevent severe large joint OA [32].

Two more byproducts of collagen breakdown have been identified, which are responsible for cartilage deformity, type II procollagen carboxy-propeptide (CPII), and urinary c-terminal crosslinking telopeptide [33]. CPII levels were upregulated in serum and SF in the early stage of OA, while the levels of CPII were found to be downregulated due to chondrocyte failure and cartilage erosion [34]. The levels of CPII were found to be directly associated with body mass index (BMI) and obesity. The underlying cause of the increased level of CPII with an increment in the level of BMI is not known, and the mechanism by which both are correlated to each other in OA is still unclear [35]. Future investigations regarding CPII involvement in OA may help to understand the pathology and prognosis of OA.

Urinary c-terminal crosslinking telopeptide of type II collagen (uCTX-II) is a catabolic product of type II collagen generated during articular cartilage degradation, excreted in SF and urine, associated with cartilage degeneration of OA [36]. Levels of uCTX-II are increased in the knee and hip OA patients and generally increased with severity but not significantly associated with radiographic knee pain. It is widely accepted to reflect cartilage erosion. The elevated level of uCTX-II is reported to predict the progression of joint space narrowing in hip OA and is also correlated to bone marrow abnormalities in disease conditions [37]. In a clinical trial, it has been found that after hyaluronic acid injection, the expression level of uCTX-II was decreased compared to baseline. Based on proteomic and bioinformatic studies, uCTX-II clusters with a biomarkers for bone metabolism [38]. It may be one of the best potential biomarkers for OA in the diagnosis, severity, monitoring of disease progression, and drug responses and is widely accepted to reflect cartilage erosion.

### 3.2. Noncollagenous Biomarkers

The extracellular matrix of joints consists of collagens, noncollagenous proteins, water, and lipids. Noncollagenous proteins include proteoglycans, cartilage oligomeric matrix protein (COMP), hyaluronan, aggrecan, fibulin, and glycoproteins such as follistatin-like protein 1 (FSTIL-1). Noncollagenous proteins are synthesized by osteoblasts and provide elasticity, strength, and flexibility to the joints. During cartilage erosion, these proteins get released into the SF that leads to OA. These proteins are reported to play the role of biomarkers and may be useful for the diagnosis and monitoring of OA progression [39].

Cartilage oligomeric matrix protein (COMP), also known as thrombospondin-5, 524 kDa homopentameric, noncollagenous glycoproteins derived from the cartilage, is found in ligaments and tendons and functions as a catalyst in collagen fibril formation [39]. During injuries and early-stage OA, COMP fragments get released into the joint which makes COMP a marker for cartilage degradation. The expression level of serum COMP was significantly elevated in the SF at the early-stage OA but surprisingly level of COMP decreased at the later stages of OA due to protease activity [40]. Also, the COMP gene mRNA level in the peripheral blood sample has been reported to be upregulated along with the COMP protein level, in the OA patients, playing a role in the structural integrity of the cartilage through interaction with extracellular matrix proteins by the cell surface integrin receptor [41]. Another study confirmed that the expression of COMP is reduced in the serum of late-stage OA due to proteolytic activity in the affected cartilage as shown in Table 2 [42]. In another study, COMP protein in OASF was downregulated in early-stage OA compared to end-stage OA and showed a positive linear correlation with age, but not significantly correlated with gender. The concentration of COMP protein in the SF increased with the severity of OA and leads to cartilage degradation [43]. Another study has found that COMP is positively and significantly correlated with IL-1*β* but negatively correlated with TNF-*α* in OA serum. The observation was reconfirmed by another study stating that COMP levels decreased with anti-IL-1*α* and IL-1*β* treatment and neutralization of IL-1*α* and IL-1*β* after the onset of disease and reduced the joint inflammation and cartilage loss [44]. The treatment of OA with mud-bath therapy (non-pharmacological approach) had an effect on pain, visual analogue scale (VAS) score, and Western Ontario and McMaster Universities Index (WOMAC) scores, but did not show any significant impact on sCOMP and other serum biomarkers except uCTX-II biomarker [45]. Increased COMP level in serum along with clinical profile may help in diagnosing and managing knee OA at the earliest possible stage.

Hyaluronan acid (HA) is the most important component of SF, provides high viscosity and smoothness to the joints and the resistance of cartilage to compression, and is associated with the radiographic progression of the disease [39]. The level of HA is significantly higher in the serum of OA, but the level decreases in SF at the late-stage of OA. The higher level of HA in serum is associated with the severity of OA as measured by higher WOMAC scores and higher Kellgrens and Lawrence (K&L) scores [46]. The K&L is a method of classifying the severity of OA using the five grading score system; grade 0-no radiological finding of X-ray of OA, grade 1-doubtful joint space narrowing (JSN) and osteophytes lipping, grade 2-definite osteophytes and JSN, grade 3-moderate multiple osteophytes, sclerosis, and possible deformity of bone ends, and grad 4-large osteophytes and severe sclerosis definite deformity of bone ends [47]. In clinical trials, injections of HA are used for the treatment of OA that increases chondrocyte synthesis of endogenous HA, prevents cartilage loss, promotes cartilage regeneration, and inhibits joint stiffness by reducing the production of proinflammatory mediators and metalloproteinases [48]. The hyaluronic acid injection is considered to be a safe procedure for the treatment of knee OA but has many side effects such as a severe inflammatory reaction at the injection site and other risk factors including an allergic reaction. Therefore, it is not commonly used but is used in severe conditions for pain relief [49]. It can be concluded that HA levels are associated with knee OA and play an important role in the identification, elevation, management, and treatment of knee OA patients.

Aggrecan: the second most important component of SF is aggrecan, known as cartilage-specific proteoglycans core protein (cspcp) or chondroitin sulfate proteoglycan. It is the major component of the extracellular matrix in cartilage along with type II collagen and plays an important role in mediating chondrocyte-chondrocyte and chondrocyte-ECM interactions in the cartilage. In OA, it is a hallmark protein for cartilage destruction [50]. In the cartilage, aggrecan is cleaved by proteolytic enzymes such as matrix metalloproteinases (MMP1) and aggrecanases, and the expression level of aggrecan gets decreased which causes cartilage destructions in OA. During in vivo studies, it has been found that aggrecan levels were high in the early stage of OA to prevent cartilage loss, but after some time, aggrecan levels get decreased due to proteolytic activity, leading to cartilage destruction. Aggrecanases were more associated with the increase of aggrecan loss associated with OA than MMP1 [51]. Thus, it can be concluded that MMPs are responsible for the normal cell aggrecan turnover, while aggrecanases contribute to its degradation under pathological conditions. In another study, when aggrecanase was inhibited, aggrecan levels gets increased, and the severity of OA gets decreased in animal models but when aggrecanase was given externally, the result was the opposite, i.e., aggrecan level gets decreased with increasing severity of OA [52]. It is indicating that there is not one protease responsible for cartilage erosion in OA but multiple proteases are responsible. Thus, rather than targeting an individual protease for OA therapy, directing research to control global protease generation may be more productive.

Fibulin peptides (Fib-3-1, Fib-3-2) are seven members of the mammalian fibulin family of glycoproteins and are associated with cartilage ECM. Basement membrane components such as proteoglycans, fibronectin, and fibrin have binding sites for Fib3-1 and Fib3-2 [53]. Specific immunoassays were developed to identify Fib3-1 and Fib3-2 in the serum and cartilage of OA patients, and their levels were increased in the serum of patients with severe knee OA compared to age-matched healthy control and were reported to be potential biomarkers of OA [54]. In another study, it has been found that increased levels of fibulin-3 epitopes are associated with middle-aged overweight, obese women, and chronic pain but are not associated with knee JSN and K&L scores [55]. Together, we can conclude that fibulin-3 epitopes make more clinical predictions than radiological features in the OA population.

Follistatin-like protein 1 (FSTIL 1) is a secreted glycoprotein, plays an important role during cell differentiation and proliferation, and is related to joint damage in OA. It is elevated in the serum, synovial tissues and SF of OA patients and is weakly expressed in the chondrocytes of the articular cartilage superficial zone in OA. In a gender-based study, it was found that level of FSTL1 was significantly higher in female OA patients compared to male patients [56]. Other studies reported that FSTL1 functions as a novel proinflammatory protein, inducing the production of cytokines TNF-*α*, IL-1*β*, IL-6, and IL-8, mediating proinflammatory events in animal models. It activates several signaling pathways including IFN-*γ* signaling, NF-K*β* pathway, p53, and p21 pathways [57]. An increase in the expression of FSTL1 induces arthritis and suppresses the production of chemokine-10 (CXCL10) and interferon-gamma (IFN-*γ*) in arthritic joints. FSTL1 is thus playing a crucial role in arthritis by inducing the IFN-*γ* signaling pathway and stimulating the molecular and cellular mechanism of innate and adaptive immune responses [58]. Several studies also provided evidence that FSTL1 can be utilized to check the severity of joint damage and is reported to be a potential biomarker for the monitoring of the progression of OA.

### 3.3. Inflammatory Biomarkers

Although the role of inflammation in OA has been heavily debated, several studies and evidence from MRI demonstrated that inflammation is also a feature of synovial joints in OA. During inflammation, macrophages and other immune cells get activated and modulate cytokine, metalloproteinase (MMPs), and complement systems [59]. The inflammatory factors are cellular factors (macrophages), molecular factors (cytokines and chemokines), and complement components that are playing a role as biomarkers and are responsible for the catabolic and anabolic destruction of the synovial joints [60]. As a part of the immune system and inflammation, the complement pathway contributes to immune responses by enhancing the action of antibodies and immune cells against antigens [61]. Activation of the complement pathways occurs due to tissue metabolism (cartilage and synovium), causing cartilage destruction and synovial inflammation, and is especially correlated with the radiographic severity of disease suggesting it as a marker for OA [62]. In OA, the concentration of complement components -C3, C5, and C9 and the complement effectors C7, C4a, and factor B were significantly higher in the SF of early OA. Factor H, C4–binding protein, C1 inhibitors, and clusterin are complement inhibitors expressed lower in OA [63]. It is found that many cartilage degradation proteins become high in the plasma and SF of OA due to activation of the complement pathway [61].

Macrophage cells are the most important cells of synovial tissues, producing aggrecanases, MMPs, and other destructive mediators such as IL-6 and TNF-*α* that are responsible for inflammatory responses. The production of these proinflammatory products depends on the NF-*κ*B signaling pathway, TLR receptors, and pathogen recognition pattern (PRR) receptors that are associated with tissue damage, inflammation, and cartilage erosion [24]. Most of the cytokines in OA joints are macrophage-mediated. Surface markers CD14 and CD163 were highly measured in plasma, serum, and SF in OA [64]. In OA, the expression of chitinase-3-like protein 1 (YKL-40), a heparin-chitin binding glycoprotein secreted from macrophage and chondrocyte during inflammation, was increased in plasma of severe OA compared to normal and mild OA [65]. Macrophages activate an innate immune response in OA pathology and progression. Thus, the products of macrophages play a role in OA pathology and can be used to understand OA mechanism and therapeutic innovations [66].

Cytokines and chemokines are small proteins secreted by immune and other cell types. Cytokines are of two types, proinflammatory: interleukin-1*β* (IL-1*β*), IL-6, IL-7, IL-15, IL-17, IL-18, TNF-*α*, or anti-inflammatory: IL-4, IL-8, and IL-10 [67]. IL-1*β* is produced by chondrocytes, osteoblasts, and synovial membrane, highly expressed in OA patients. IL-1 increases the expression of MMPs and nitric oxide (NO) expression, inhibits the synthesis of both proteoglycan and collagen, and thus promotes inflammatory responses [68]. The catabolic effect on synovium tissue is due to activation of Wnt target gene transcription by decreasing the expression of dickkopf the WNT signaling pathway inhibitor 1 (DKK1) and secreted frizzled-related protein 3 precursor (FRZB) [69]. Levels of proinflammatory cytokines IL-6 and TNF-*α* were elevated in OASF. Expression levels of TNF-*α* were highly increased in OA patients but soluble TNF-*α* receptor levels were found to be lower in OASF and plasma [70]. Increased levels of IL-6 and TNF-*α* are associated with JSN that induces cartilage loss by activating NF-*κ*B and MAPK signaling pathways. Activated NF-*κ*B upregulates the expression of chemokines, cytokines, and many other growth factors which lead to an imbalance in the homeostasis of the subchondral bone [71]. The expression level of IL-6 can be induced by IL-1*β* in chondrocytes during inflammation that suppresses type-II collagen activity, leading to cartilage degeneration and an increase in the high level of IL-6 in OASF [72]. IL-7 is a hematopoietic growth factor secreted by the stromal cells, considered to be a proinflammatory factor, highly expressed in OA synovium, and involved in regulating bone homeostasis [73]. IL-4 and IL10 are anti-inflammatory cytokines, and levels were found to be reduced in OA plasma, suppressing bone resorption in OA. IL-4 inhibits the expression of IL-1, IL-6, MMP13, TNF-*α*, and receptor activator of nuclear factor *β* ligand (RANKL) in the cells that modulate osteoclast proliferation [74].

**Figure 3:**
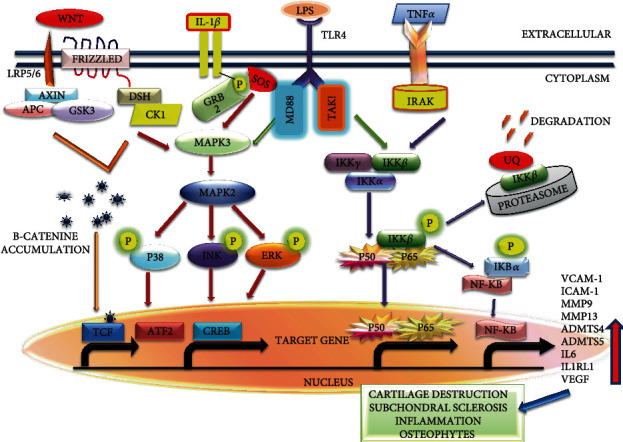
Biomarkers play role in pathology of OA by multiple signaling pathways including NF-*κ*B pathway, MAPK pathway, Wnt-signaling pathway, and TLR pathway. Wnt: wingless and Int-1; APC: adenomatous polyposis coli; GSK3*β*: glycogen synthese kinase; DSH: phosphoprotein disheveled; IL-1*β*: interleukin 1 beta; GRB2: growth factor receptor-bound protein 2; MAPK: mitogen-activated protein kinase; ERK: extracellular signal-regulated kinase; JNK: c-Jun N-terminal kinase; TLR4: toll-like receptor 4; MD88: myeloid differentiation primary response 88; IKK: inhibitor of nuclear factor-*κ*B (I*κ*B) kinase; NF-*κ*B: nuclear factor kappa light chain enhancer of activated B cells; IRAK: IL-1 receptor-associated kinase; P: phosphorylation; UQ: ubiquitin; CREB: cAMP-responsive element binding protein; TCF: T cell factor; TAK1: transforming growth factor b-activated kinase 1; MMP: matrix metallopeptidases; VCAM-1: vascular cell adhesion protein; ICAM-1: intercellular adhesion molecule 1.

Other inflammatory markers and chemokines are small molecular weight and cell signaling proteins that attract cells via chemotaxis. These are secreted during the immune response and regulate the migration of cells during the development of immune response [75]. Chemokines such as CCL14, CCL19, CCL20, CCL21, CXC27, and CXCL12 are responsible for homeostasis while CXC8, CCL2, CCL3, CCL5, and CXCL10 are partners in the inflammatory response [76]. The levels of chemokines were high in the plasma and SF of OA patients, causing cartilage loss and inflammation in the synovial membrane [77]. Thus, inflammatory mediators are playing a role in OA progression by activating several signaling pathways, including the NF-*κ*B signaling pathway, the wnt signaling pathway, the TLR-4 receptor, and the MAPK signaling pathway [78]. They can be used to understand mechanisms and pathways responsible for OA progression and help in the discovery of new drugs by targeting their specific receptors as shown in Figure 3.

### 3.4. Posttranslational Modifications (PTM) in Extracellular Matrix Molecules

In cartilage, posttranslational modifications (PTMs) in proteins can enhance the proteome complexity by altering the proteins involved in enzymatic, biochemical, and physiological processes affecting OA pathogenesis. Therefore, identification and understanding of PTMs are important in OA disease treatment and prevention [79]. Posttranslational modifications can be grouped into natural which include methionine, oxidation, phosphorylation, ubiquitination, deamidation, and synthetic subgroups. Nonenzymatic PTMs such as the deamidated epitope of cartilage oligomeric matrix protein (D-COMP) and pentosidine can be used to examine deficiencies in extracellular matrix macromolecules in OA [80].

The deaminated epitope of COMP (Asn^64^ converted into Asp^64^) is known as D-COMP. A comparative study between D-COMP and total native COMP in the patients undergoing joint replacement surgery has shown increased expression levels of serum D-COMP as well as a decline D-COMP after replacement of joint tissue in knee OA [80]. Expression levels of D-COMP were found to be significantly higher in hip cartilage lesion extracts than in knee OA lesions. Hence, D-COMP in cartilage and systemic circulation was reported to be the first biomarker for specific joint tissue sites. In contrast, COMP was associated with radiographic knee OA but not associated with hip OA severity [81]. Thus, the PTMs in COMP may be related to the progression of OA in different joints.

Pentosidine is a biomarker for advanced glycated end products (AGEs), rapidly produced under various types of oxidative stress and hyperglycemia in articular cartilage, serum, urine, and SF [82]. Pentosidine levels were mostly increased in inflammation conditions, associated with OA. The expression level of urinary pentosidine levels was also found to be elevated in patients with hand, knee, and hip OA. Urinary pentosidine levels were related to the measurements of joint pain, stiffness, and disability in patients with erosive hand OA [83]. Urine pentosidine positively correlated with both C-reactive protein (CRP) and erythrocyte sedimentation rate (ESR) levels during oxidative stress. An increased level of pentosidine was also found in disease conditions associated with increased oxidative stress. Nonenzymatic glycation of proteins was also associated with aging, increased cartilage stiffness, increased degradation of ECM proteins, and decreased proteoglycans synthesis by chondrocytes [84]. The accumulation of pentosidine in cartilage was reported to be an etiologic factor for the development and progression of OA. In conclusion, high levels of pentosidine in OA and RA patients indicate that this compound is not only a marker of glycoxidation but may be used as a more general marker of oxidative stress in different pathologies inluding OA [85].

### 3.5. Other Biomarkers

Several aggrecanase enzymes (a disintegrin and metalloproteinase with thrombospondin motifs ADAMTS) and proteolytic enzymes such as metalloproteinases (MMPs) and obesity-related proteins are associated with cartilage degradation and are identified as OA biochemical markers [86].

A disintegrin and metalloproteinase with thrombospondin motifs (ADAMTS), a family of secreted metalloproteinase, involved in various developmental and homeostatic processes. This ADAMTS degrades type II collagen and aggrecan and is associated with the progression of OA [87]. The ADAMTS-1 expression was significantly upregulated in OA cartilage but showed reduced expression in late-stage OA. Immunohistochemistry analysis indicated that in the normal cartilage, ADAMTS-1 was predominantly expressed in the superficial zone, whereas the central zone and osteophytes showed increasing levels of expression of ADAMTS [88]. ADAMTS-2, 3, and 14 are procollagen N-proteinases responsible for the removal of the N-terminal propeptide of type I, II, III, and V procollagen and thereby the formation of the collagen fibrils. The expression levels of ADAMTS-2,-3, and -14 were found to be significantly upregulated in the OA cartilage. Single nucleotide polymorphisms in the ADAMTS-14 gene were reported to be associated with an increased risk of knee OA in two female cohorts [89]. The two “aggrecanases” ADAMTS-4 and -5 degrade the aggrecan and collagen, and their degradation was correlated with the progression of OA [90]. ADAMTS-7 and 12 were found to contribute to OA pathogenesis by degrading COMP. The degradation of COMP can be inhibited in vitro by adding ADAMTS-7 neutralizing antibodies or small interfering RNA (siRNA) [91]. ADAMTS-9 was expressed in normal cartilage and was induced in response to proinflammatory cytokines and adipokines and was found to be reduced in late-stage OA cartilage [92]. ADAMTSs bind with cytokines, growth factor precursors, and their cytoplasmic domain, activate intracellular signaling cascades, and stimulate secretions of MMPs, IL-6, TNF-*α*, and other proinflammatory markers. Therefore, ADMTS-4 and -5 and many other ADAMTs can be used to understand the pathophysiology of OA and to enable targeted inhibitors for cartilage repair [93].

Adipokines: the adipocyte-derived molecules “adipokines” also play an important role in cartilage and bone homeostasis in OA. The association of adipokines with obesity, having both properties, pro- or anti-inflammatory properties, suggests that adipokines are an important mediator that links inflammation with obesity and OA [94]. Adipokines such as leptin, adiponectin, visfatin, and resistin play an important role in OA and are used as markers for OA. All of the three adipokines (leptin, visfatin, and resistin) were elevated in OA plasma while adiponectin was increased in OA SF [95]. Leptin induces chondrocytes to secrete cartilage degradation mediators such as TNF-*α*, IL-1*β*, IL-6, IL-8, reduced proliferation of chondrocytes, increased osteoblast proliferation, and ossification of cartilage, increasing inflammation in OA [96]. Leptin stimulates cytokine secretion and proliferation of T-cell through the MAPK and phosphatidylinositol-3 kinase (PIK3) pathway. It thus induces two different markers early activation marker (CD69) and late activation marker (CD25 and CD71) in T lymphocyte cell that leads to inflammation in synovial joints [97]. Other adipokines such as adiponectin that has both catabolic and anabolic effects provide a proinflammatory function by stimulating NOS2, MCP-1, MMP-1, MMP-3, MMP-9, MMP-13, IL-6, IL-8, PGE2, and vascular endothelial growth factor (VEGF) [98]. Adiponectin stimulates the release of anti-inflammatory molecules (IL-10 and IL-1) receptor antagonists, suggesting a protective role against cartilage damage. Besides, adiponectin has been shown to increase chondrocyte proliferation, aggrecan synthesis, matrix mineralization, and upregulated type II and type X collagen expression [99].

In order to understand the role of obesity in OA development, an association of leptin and adiponectin with adiposity and OA was evaluated and found that leptin was strongly associated with knee OA compared to hand OA but adiponectin was not associated with either knee and hand OA [100]. In vitro and in vivo studies have shown that leptin receptors are overexpressed in the cartilage and osteophytes in OA providing further support that leptin signaling is essential for obesity-induced OA development [101, 102]. The expression levels of visfatin and resistin were significantly upregulated in erosion hand OA (E-HOA) compared to normal controls (NC). The expression of the resistin was also higher in nonerosive hand OA (NE-HOA) than in controls, but the level of the visfatin was only higher in E-HOA. So, these result showed that visfatin is a good biomarker to distinguish the difference between E-HOA and NE-HOA. Furthermore, the high level of resistin in serum suggests a possible role in OA pathophysiology [103]. Another study was performed to understand the effect of adipokines on microRNAs (miRNA), MMPs, and collagen type II alpha 1 chains (Col2a1) in OA pathology. OA chondrocyte cells were stimulated with visfatin and resistin at optimal concentration. It was found that the expression levels of miRNA (34a, 155, 181a, let7e), MMP1, and MMP13 were significantly higher while miRNA-140 and Col2a1 were downregulated in the cartilage. Increased levels of miRNA play a role in cartilage breakdown, cell differentiation and proliferation, production of inflammatory cytokines, and cartilage homeostasis [104].

Retinol binding protein (RBP-4) is an adipocyte-derived factor and a member of the lipocalin family that plays a role as a vitamin A carrier in the blood and is functionally involved in insulin resistance (type 2 diabetes) and metabolic syndrome (MetS). The expression level of RBP4 was found to be significantly upregulated in plasma, SF, cartilage tissue, and chondrocytes of OA. In cartilage tissue of OA, RBP4 is positively associated with other adipokines (adipsin, leptin, and resistin), metalloproteases (MMP1 and MMP3), and proinflammatory protein YKL-40. RBP4 induces the expression of inflammatory and catabolic factors in OA disease by activating stimulated by retinoic acid gene homolog 6′ (STRA6) receptor and TLR-4 signaling pathway [105]. But out of them, no individual adipokine has been reported to be used in the diagnosis of OA. In conclusion, it can be said that adipokines play an important role in OA progression and pathology. For the treatment of OA, specific adipokines may be useful to develop a therapeutic molecule that targets specific adipokines [106].

## 4. Omic Approaches for Identification of Biomarkers

Different techniques and methods of proteomics approaches are used for the identification of proteins, their activity, and presence as biomarkers in disease conditions compared to control from different samples [107]. For the discovery of new biomarkers, these approaches include some essential steps as shown in Figure 1: extraction and separation of proteins, identification of proteins, and validation of proteins. Finally, the discovery of a new biomarker requires clinical trials [108].

Proteins are extracted and separated by two-dimension gel electrophoresis (2-DE), and proteins are separated based on isoelectric points and molecular weights for the ability to compare the number of proteins and isoforms in the same gels [109]. Two-dimensional intergel electrophoresis (2D-DIGE) is also used to extract and separate proteins and is more reliable than 2-DE because it provides greater sensitivity and reproducibility. In this method, different types of fluorescent dyes are used for the labeling of proteins, and these signals can also be used to identify proteins at the same spots in the gel [110]. There are different proteomics techniques used for the identification of proteins including matrix-assisted laser desorption/ionization time-of-flight (MALDI-TOF), liquid chromatography with tandem mass spectroscope (LC-MS/MS), surface-enhanced laser desorption/ionization-time-of-flight (SELDI-TOF), selected reaction monitoring (SRM), and multiple reaction monitoring (MRM) [111, 112].

High-throughput quantitative techniques like proteomics analysis have also been utilized for the discovery of lower abundance ECM components, lipoproteins, and complement components in OA serum and SF to identify novel diseases relevant to OA biomarkers. The MALDI-TOF and LC-MS/MS are used to determine the differentially expressed proteins in OA [113]. Many proteins have been identified in the different biological fluids such as blood, urine, SF, and tissue [114]. Besides, ingel digestion and reverse-phase peptide separation were used in the liquid chromatography system. The majority of the proteins identified are involved in inflammation (COMP), complement activation (C3-C4), and immune responses (MMP) [114]. It can therefore be concluded that proteomics analysis followed by MALDI and imaging mass spectroscopy (IMS) is used to identify differentially expressed proteins to specific anatomic areas of joint tissues of OA patients and is a powerful technique to examine the photomicrograph of the selection before the usage of MALDI [115]. In a comparative study of protein patterns from OA and non-OA, cartilage tissues were digested with metalloproteases enzyme, released breakdown products of cartilage tissue, type II collagen, fibronectin, COMP, and aggrecan and were monitored by LC-MS/MS [116].

The SELDI-TOF technique was used to analyze SF samples from OA patients, a chromatographic surface that can bind with the specific site of interested protein based on electrostatic interaction, absorption, and biochemical affinity. The result demonstrated that three mass peaks were identified as potential markers (3893, 10576, and 14175 Da), to differentiate OA. Out of them, the peak 10576 Da was identified as S100 calcium-binding protein with a sensitivity of 89.4% and specificity of 91.2% [117]. Selected reaction monitoring (SRM) and multiple reaction monitoring (MRM) were used to validate differentially expressed proteins such as the dickkopf WNT signaling pathway inhibitor 3 (DKK3) in the SFof control and OA [118]. Several proteomic techniques are available for the validation of these identified proteins, including western blotting and enzyme-linked immunosorbent assay (ELISA). Expression of these biomarkers including collagen breakdown products (type II collagen, PIIANP, and CPII), noncollagenous proteins (COMP, aggrecan, fibulin 3-1, and FSTL-1), and inflammatory biomarkers (IL-1*β*, TNF-*α*, and cytokines) was monitored by western blotting and ELISA in the different biological fluid (serum, urine, synovial fluid, and tissue) of OA and control [40, 46, 54, 60]. To understand the role of these biomarkers in OA pathophysiology, bioinformatic tools are used to monitor physiological connection and their interactions with other proteins [119]. Bioinformatic tools such as GO annotation, DAVID, KEGG pathway, and STRING analysis were used to analyze omics data, function analysis, pathway analysis, protein-protein interactions, and interpretation of data [120].

Other omic-based approaches such as genomics and metabolomics approaches are used to understand disease mechanisms and identify biomarkers for the discovery of therapeutic and clinical development. [121]. The genomic approaches were used to identify genetic mutations (whole genome), genetic variations with disease condition using genome-wide association studies (GWAS), identification of differentially expressed genes (DEGs) by gene expression omnibus (GEO), and m-RNA and mi-RNA expression by microarray to compare between control and disease conditions [122–124]. The identified gene (COMP gene) was used for disease diagnosis, prognosis, and therapeutic efficiency [41]. The metabolomic approach has the ability to detect metabolites from biological fluids using nuclear magnetic resonance (NMR) and mass spectroscope (MS) that detect biomarkers for early-stage diagnosis of OA [125]. The expression level of arginine metabolites was found to be decreased in the plasma of OA patients, leading to an imbalance between cartilage erosion and repair [126]. Other metabolites such as taurine and L-carnitine has been reported to be involved in the pathogenesis of subchondral sclerosis [127]. Metabolic arrays are used to detect early-stage biomarkers and their identity to monitor changes in disease status [125]. Thus, these proteomic approaches are promising approaches for identifying reliable biomarkers, can be used to diagnose disease, monitor disease progression, and discover new therapeutic drugs.

## 5. Conclusion

As seen from the presented literature, there are many challenges in interpreting the results of biomarker studies. However, the main limitation is the specificity of the biomarker for a particular outcome in OA, particularly as there is a paucity of reference measures usable in OA and notably sensitive to change. The biomarker must be highly specific and sensitive to OA joints that can be used for diagnosis, prognosis, and treatment of OA. Currently, joint pain, stiffness, and joint deformity that are the hallmarks of OA initiation are monitored by radiographic analysis. Special diagnostic tools and treatments for OA rarely exist; so, this problem could be resolved by identifying some reliable biomarkers that can be widely implemented in clinical settings. In the case of OA disease, tissues undergo metabolic changes as well as structural and morphological changes. Several biomarkers related to metabolic changes can provide valuable information for the diagnosis and development of new drugs for the treatment of OA. This review demonstrates that the imbalance of degradation and synthesis of cartilage disrupts by multiple factors, including aging and mechanical loading on the joints. Beside this, the involvement of different signaling pathways can disrupt the balance between catabolic and anabolic activities in cartilage that results in degradation of proteoglycan and ECM component. Manipulation of the abovementioned molecules in particular chondrocytes could also play a role in articular cartilage regeneration. Further, studies will explore these biomarkers with clinical settings, be used for the diagnosis of OA at an early stage, and help in the treatment of OA cost-effectively by discovering new therapeutic drugs. Beyond the aspects, there is a need for awareness of a different kind of molecular mechanisms involved in OA onset and progress, which could stimulate understanding about early diagnosis and therapeutic intervention. Novel proteomic techniques and approaches as well as new application are needed to accomplish the identification of specific protein as a biomarker for diagnosis and treatment of OA. As a final remark, novel diagnostic markers are urgently needed to improve the prognosis of OA patients.

## Figures and Tables

**Table 1:** The selected biomarkers of OA are classified according tissue involvement and metabolism.

Categories	Candidate biomarkers with reference
Cartilage degradation biomarkers	C2C [34], Coll2, Coll2-1, CoLL2-1NO [27], CTX-II [36], COMP [41], YKL-40 [65]
Cartilage synthesis biomarkers	uCTX [36], PIIANP [31], PIIACP [32]
Synovial degradation biomarkers	HA [48], fibuline-3 [53], follistatin-like protein 1(FSTL1) [56]
Synovial synthesis biomarkers	sPIIINP [31]
Extracellular matrix biomarkers	ARGS [50], MMPs [98]
Inflammatory biomarkers	Complement components [63], cytokines (IL-1*β*, TNF-*α*, IL-4, IL-7, IL-8, IL-10) [68–73], and chemokines [75]
Posttranslation modification biomarkers	DCOMP [81], pentosidine [83]
Other biomarkers	ADMTS [88], obesity-related protein, and adipokines [94]

CTXII: type II collagen; C2C: type II collagen cleavage product; Coll 2: type II collagen, NO: nitrogen oxide; COMP: cartilage oligomeric matrix protein; YKL-40: chitinase 3-like protein 1; PIIANP: type IIA collagen N-propeptide; PIICP: type II C propeptide; *μ*CTX-1: urinary c-terminal crosslinking telopeptide II collagen; HA: hyaluronic acid; sPIIINP: serum III procollagen; FIB3-1: fibulin-3 peptides; ARGS: aggrecanase-cleaved aggrecan; MMPs: matrix metalloproteinases; IL-1*β*: interleukin 1 beta; TNF-*α*: tumour narcosis factor-alpha.

**Table 2:** List of biomarkers routinely used in the diagnosis and treatment of osteoarthritis and the studies of these markers in patients.

Biomarker name	Description	Evidence for role in OA	Sample type	Expression level in OA	References
Coll-2	Type-II collagen	Cartilage loss	S, SF	Upregulated	[28]
PIIANP	Type IIA collagen N-propeptide	Proanabolic agent of cartilage	S, SF	Downregulated	[32]
CPII	Type II procollagen carboxy propeptide	Destruction and loss of cartilage	S, SF	Downregulated	[34]
COMP	Cartilage oligomeric matrix protein	Cartilage loss	S, SF	Upregulated in an early stage, downregulated in late stage	[40]
HA	Hyaluronic acid	Immobility, cartilage loss, and stiffness in joints	S, SF	Downregulated	[46]
Aggrecan		Loss connectivity between chondrocyte-ECM	S, SF	Downregulated	[51]
FSTL1	Follistatin-like protein	Induces IFN-*γ* signaling pathway and stimulates proinflammatory factors	S, SF, U	Upregulated	[56]
IL-1	Interleukin -1	Promotes activation of chondrocyte osteoclasts. Proinflammatory cytokines	S, SF	Upregulated	[68]
IL-6	Interleukin-6	Promotes neutrophil chemotaxis and proinflammatory cytokines	S,SF	Upregulated	[70]
D-COMP	Deamidation of COMP	Aging and progression of hip OA	S	Upregulated	[80]
Pentosidine	Advanced glycation end products	Degradation of ECM proteins and decrease proteoglycans synthesis	S, U	Upregulated	[83]
ADAMTs	A disintegrin and metalloproteinases	Cartilage degradation	Cartilage tissue	Upregulated	[89]
Adipokines (leptin visfatin, resistin, adiponectin)	Adipose tissue related proteins	Cartilage loss and degradation of ECM protein	S, SF	Upregulated except Adiponectin	[96]

S: serum; SF: synovial fluid; U: urine.

## Additional Points

*Highlights.* (1) OA is a cartilage degenerative disease with an unknown molecular mechanism. (2) Biomarkers can be used for OA diagnosis and therapeutic drug discovery. (3) Most of the biomarker are the result of metabolic changes of tissue such as cartilage, bone, and synovium metabolism.

## References

[1] Loeser R. F., Goldring S. R., Scanzello C. R., Goldring M. B. (2012). Osteoarthritis: a disease of the joint as an organ. *Arthritis and Rheumatism*.

[2] March L., Cross M., Lo C. (2016). *Osteoarthritis: a serious disease*.

[3] Safiri S., Kolahi A. A., Hoy D. (2019). Global, regional and national burden of rheumatoid arthritis 1990–2017: a systematic analysis of the Global Burden of Disease study 2017. *Annals of the Rheumatic Diseases*.

[4] Cui A., Li H., Wang D., Zhong J., Chen Y., Lu H. (2020). Global, regional prevalence, incidence and risk factors of knee osteoarthritis in population-based studies. *EClinicalMedicine*.

[5] Deshpande B. R., Katz J. N., Solomon D. H. (2016). Number of persons with symptomatic knee osteoarthritis in the US: impact of race and ethnicity, age, sex, and obesity. *Arthritis Care & Research*.

[6] Pal C. P., Singh P., Chaturvedi S., Pruthi K. K., Vij A. (2016). Epidemiology of knee osteoarthritis in India and related factors. *Indian Journal of Orthopaedics*.

[7] Palazzo C., Nguyen C., Lefevre-Colau M. M., Rannou F., Poiraudeau S. (2016). Risk factors and burden of osteoarthritis. *Annals of Physical and Rehabilitation Medicine*.

[8] Conaghan P. G., Felson D., Gold G., Lohmander S., Totterman S., Altman R. (2006). MRI and non-cartilaginous structures in knee osteoarthritis. *Osteoarthritis and Cartilage*.

[9] Pelletier J. P., Cooper C., Peterfy C. (2013). What is the predictive value of MRI for the occurrence of knee replacement surgery in knee osteoarthritis?. *Annals of the Rheumatic Diseases*.

[10] Yasuda T. (2014). Cartilage destruction by matrix degradation products. *Modern Rheumatology*.

[11] Bobinac D., Spanjol J., Zoricic S., Maric I. (2003). Changes in articular cartilage and subchondral bone histomorphometry in osteoarthritic knee joints in humans. *Bone*.

[12] Pastrama M. I., Ortiz A. C., Zevenbergen L. (2019). Combined enzymatic degradation of proteoglycans and collagen significantly alters intratissue strains in articular cartilage during cyclic compression. *Journal of the Mechanical Behavior of Biomedical Materials*.

[13] Wilson N., Sanchez-Riera L., Morros R. (2015). Drug utilization in patients with OA: a population-based study. *Rheumatology*.

[14] Kraus V. B., Burnett B., Coindreau J. (2011). Application of biomarkers in the development of drugs intended for the treatment of osteoarthritis. *Osteoarthritis and Cartilage*.

[15] Hong J. I., Park I. Y., Kim H. A. (2020). Understanding the molecular mechanisms underlying the pathogenesis of arthritis pain using animal models. *International Journal of Molecular Sciences*.

[16] Bay-Jensen A. C., Reker D., Kjelgaard-Petersen C. F. (2016). Osteoarthritis year in review 2015: soluble biomarkers and the BIPED criteria. *Osteoarthritis and Cartilage*.

[17] Dinçel Y. M. (2018). Value of biomarkers in osteoarthritis. *Osteoarthritis Biomarkers and Treatments*.

[18] Van Spil W. E., Szilagyi I. A. (2020). Osteoarthritis year in review 2019: biomarkers (biochemical markers). *Osteoarthritis and Cartilage*.

[19] Kraus V. B., Blanco F. J., Englund M. (2015). OARSI clinical trials recommendations: soluble biomarker assessments in clinical trials in osteoarthritis. *Osteoarthritis and Cartilage*.

[20] Selleck M. J., Senthil M., Wall N. R. (2017). Making meaningful clinical use of biomarkers. *Biomarker Insights*.

[21] Watt F. E. (2018). Osteoarthritis biomarkers: year in review. *Osteoarthritis and Cartilage*.

[22] Roberts H. M., Law R. J., Thom J. M. (2019). The time course and mechanisms of change in biomarkers of joint metabolism in response to acute exercise and chronic training in physiologic and pathological conditions. *European Journal of Applied Physiology*.

[23] Lotz M., Martel-Pelletier J., Christiansen C. (2014). Republished: value of biomarkers in osteoarthritis: current status and perspectives. *Postgraduate Medical Journal*.

[24] Chen Y., Jiang W., Yong H. (2020). Macrophages in osteoarthritis: pathophysiology and therapeutics. *American Journal of Translational Research*.

[25] Birmingham J. D., Vilim V., Kraus V. B. (2006). Collagen biomarkers for arthritis applications. *Biomarker Insights*.

[26] Garnero P., Ayral X., Rousseau J. C. (2002). Uncoupling of type II collagen synthesis and degradation predicts progression of joint damage in patients with knee osteoarthritis. *Arthritis and Rheumatism*.

[27] Deberg M., Labasse A., Christgau S. (2005). New serum biochemical markers (Coll 2-1 and Coll 2-1 NO_2_) for studying oxidative-related type II collagen network degradation in patients with osteoarthritis and rheumatoid arthritis. *Osteoarthritis and Cartilage*.

[28] Mobasheri A., Lambert C., Henrotin Y. (2019). Coll2-1 and Coll2-1NO2 as exemplars of collagen extracellular matrix turnover – biomarkers to facilitate the treatment of osteoarthritis?. *Expert Review of Molecular Diagnostics*.

[29] Shi S., Man Z., Li W., Sun S., Zhang W. (2016). Silencing of Wnt5a prevents interleukin-1*β*-induced collagen type II degradation in rat chondrocytes. *Experimental and Therapeutic Medicine*.

[30] Henrotin Y., Chevalier X., Deberg M. (2013). Early decrease of serum biomarkers of type II collagen degradation (Coll2-1) and joint inflammation (Coll2-1 NO2) by hyaluronic acid intra-articular injections in patients with knee osteoarthritis: a research study part of the Biovisco study. *Journal of Orthopaedic Research*.

[31] Sugiyama S., Itokazu M., Suzuki Y., Shimizu K. (2003). Procollagen II C propeptide level in the synovial fluid as a predictor of radiographic progression in early knee osteoarthritis. *Annals of the Rheumatic Diseases*.

[32] Daghestani H. N., Jordan J. M., Renner J. B., Doherty M., Wilson A. G., Kraus V. B. (2017). Serum N-propeptide of collagen IIA (PIIANP) as a marker of radiographic osteoarthritis burden. *PLoS One*.

[33] Boeth H., Raffalt P. C., Mac Mahon A. (2019). Association between changes in molecular biomarkers of cartilage matrix turnover and changes in knee articular cartilage: a longitudinal pilot study. *Journal of Experimental Orthopaedics*.

[34] Conrozier T., Poole A. R., Ferrand F. (2008). Serum concentrations of type II collagen biomarkers (C2C, C1, 2C and CPII) suggest different pathophysiologies in patients with hip osteoarthritis. *Clinical and Experimental Rheumatology*.

[35] Cahue S., Sharma L., Dunlop D. (2007). The ratio of type II collagen breakdown to synthesis and its relationship with the progression of knee osteoarthritis. *Osteoarthritis and Cartilage*.

[36] Chmielewski T. L., Trumble T. N., Joseph A. M. (2012). Urinary CTX-II concentrations are elevated and associated with knee pain and function in subjects with ACL reconstruction. *Osteoarthritis and Cartilage*.

[37] Huang M., Zhao J., Huang Y., Dai L., Zhang X. (2018). Meta-analysis of urinary C-terminal telopeptide of type II collagen as a biomarker in osteoarthritis diagnosis. *Journal of Orthopaedic Translation*.

[38] Klocke R., Levasseur K., Kitas G. D., Smith J. P., Hirsch G. (2018). Cartilage turnover and intra-articular corticosteroid injections in knee osteoarthritis. *Rheumatology International*.

[39] Garvican E. R., Vaughan-Thomas A., Clegg P. D., Innes J. F. (2010). Biomarkers of cartilage turnover. Part 2: Non-collagenous markers. *The Veterinary Journal*.

[40] Verma P., Dalal K. (2013). Serum cartilage oligomeric matrix protein (COMP) in knee osteoarthritis: a novel diagnostic and prognostic biomarker. *Journal of Orthopaedic Research*.

[41] Arellano R. D., Aguilar L. S., Argüello R., Hernadez F., Gonzalez F. F., Moran J. (2017). Cartilage oligomeric matrix protein levels in synovial fluid in patients with primary knee osteoarthritis and healthy controls: a preliminary comparative analysis with serum cartilage oligomeric matrix protein. *Archives of Rheumatology*.

[42] Riegger J., Rehm M., Büchele G. (2020). Serum cartilage oligomeric matrix protein in late-stage osteoarthritis: association with clinical features, renal function, and cardiovascular biomarkers. *Journal of Clinical Medicine*.

[43] Spakova T., Harvanova D., Lacko M. (2020). A preliminary study of combined detection of COMP, TIMP-1, and MMP-3 in synovial fluid: potential indicators of osteoarthritis progression. *Cartilage*.

[44] Sharif M., Kirwan J. R., Elson C. J., Granell R., Clarke S. (2004). Suggestion of nonlinear or phasic progression of knee osteoarthritis based on measurements of serum cartilage oligomeric matrix protein levels over five years. *Arthritis and Rheumatism*.

[45] Pascarelli N. A., Cheleschi S., Bacaro G., Guidelli G. M., Galeazzi M., Fioravanti A. (2016). Effect of mud-bath therapy on serum biomarkers in patients with knee osteoarthritis: results from a randomized controlled trial. *The Israel Medical Association Journal: IMAJ*.

[46] Filková M., Šenolt L., Braun M. (2009). Serum hyaluronic acid as a potential marker with a predictive value for further radiographic progression of hand osteoarthritis. *Osteoarthritis and Cartilage*.

[47] Luijkx T., Pai V. (2016). Kellgren and Lawrence system for classification of osteoarthritis of knee. *Datum Pristupa*.

[48] Bannuru R. R., Natov N. S., Obadan I. E., Price L. L., Schmid C. H., McAlindon T. E. (2009). Therapeutic trajectory of hyaluronic acid versus corticosteroids in the treatment of knee osteoarthritis: a systematic review and meta-analysis. *Arthritis Care and Research*.

[49] Bowman S., Awad M. E., Hamrick M. W., Hunter M., Fulzele S. (2018). Recent advances in hyaluronic acid based therapy for osteoarthritis. *Clinical and Translational Medicine*.

[50] Dudhia J. (2005). Aggrecan, aging and assembly in articular cartilage. *Cellular and Molecular Life Sciences*.

[51] Sandy J. D., Verscharen C. (2001). Analysis of aggrecan in human knee cartilage and synovial fluid indicates that aggrecanase (ADAMTS) activity is responsible for the catabolic turnover and loss of whole aggrecan whereas other protease activity is required for C-terminal processing in vivo. *The Biochemical Journal*.

[52] Sandy J. D. (2006). A contentious issue finds some clarity: on the independent and complementary roles of aggrecanase activity and MMP activity in human joint aggrecanolysis. *Osteoarthritis and Cartilage*.

[53] Timpl R., Sasaki T., Kostka G., Chu M. L. (2003). Fibulins: a versatile family of extracellular matrix proteins. *Nature Reviews. Molecular Cell Biology*.

[54] Henrotin Y., Gharbi M., Mazzucchelli G., Dubuc J. E., De Pauw E., Deberg M. (2012). Fibulin 3 peptides Fib3-1 and Fib3-2 are potential biomarkers of osteoarthritis. *Arthritis and Rheumatism*.

[55] Runhaar J., Sanchez C., Taralla S., Henrotin Y., Bierma-Zeinstra S. M. (2016). Fibulin-3 fragments are prognostic biomarkers of osteoarthritis incidence in overweight and obese women. *Osteoarthritis and Cartilage*.

[56] Wang Y., Li D., Xu N. (2011). Follistatin-like protein 1: a serum biochemical marker reflecting the severity of joint damage in patients with osteoarthritis. *Arthritis Research & Therapy*.

[57] Ni S., Miao K., Zhou X. (2015). The involvement of follistatin-like protein 1 in osteoarthritis by elevating NF-*κ*B-mediated inflammatory cytokines and enhancing fibroblast like synoviocyte proliferation. *Arthritis Research & Therapy*.

[58] Li W., Alahdal M., Deng Z. (2020). Molecular functions of FSTL1 in the osteoarthritis. *International Immunopharmacology*.

[59] Woodell-May J. E., Sommerfeld S. D. (2019). Role of inflammation and the immune system in the progression of osteoarthritis. *Journal of Orthopaedic Research*.

[60] Daghestani H. N., Kraus V. B. (2015). Inflammatory biomarkers in osteoarthritis. *Osteoarthritis and Cartilage*.

[61] Silawal S., Triebel J., Bertsch T., Schulze-Tanzil G. (2018). Osteoarthritis and the complement cascade. *Clinical Medicine Insights: Arthritis and Musculoskeletal Disorders*.

[62] Wang Q., Rozelle A. L., Lepus C. M. (2011). Identification of a central role for complement in osteoarthritis. *Nature Medicine*.

[63] Struglics A., Okroj M., Swärd P. (2016). The complement system is activated in synovial fluid from subjects with knee injury and from patients with osteoarthritis. *Arthritis Research & Therapy*.

[64] Daghestani H. N., Pieper C. F., Kraus V. B. (2015). Soluble macrophage biomarkers indicate inflammatory phenotypes in patients with knee osteoarthritis. *Arthritis & Rheumatology*.

[65] Väänänen T., Koskinen A., Paukkeri E. L. (2014). YKL-40 as a novel factor associated with inflammation and catabolic mechanisms in osteoarthritic joints. *Mediators of Inflammation*.

[66] Zhang H., Cai D., Bai X. (2020). Macrophages regulate the progression of osteoarthritis. *Osteoarthritis and Cartilage*.

[67] Hanada T., Yoshimura A. (2002). Regulation of cytokine signaling and inflammation. *Cytokine & Growth Factor Reviews*.

[68] Attur M., Belitskaya-Lévy I., Oh C. (2011). Increased interleukin-1*β* gene expression in peripheral blood leukocytes is associated with increased pain and predicts risk for progression of symptomatic knee osteoarthritis. *Arthritis and Rheumatism*.

[69] Zhong L., Schivo S., Huang X., Leijten J., Karperien M., Post J. N. (2017). Nitric oxide mediates crosstalk between interleukin 1*β* and WNT signaling in primary human chondrocytes by reducing DKK1 and FRZB expression. *International Journal of Molecular Sciences*.

[70] Simão A. P., de Oliveira Almeida T. M., Mendonça V. A. (2014). Soluble TNF receptors are produced at sites of inflammation and are inversely associated with self-reported symptoms (WOMAC) in knee osteoarthritis. *Rheumatology International*.

[71] Choi M. C., Jo J., Park J., Kang H. K., Park Y. (2019). NF-*κ*B signaling pathways in osteoarthritic cartilage destruction. *Cell*.

[72] Stannus O., Jones G., Cicuttini F. (2010). Circulating levels of IL-6 and TNF-*α* are associated with knee radiographic osteoarthritis and knee cartilage loss in older adults. *Osteoarthritis and Cartilage*.

[73] van Roon J. A., Lafeber F. P. (2008). Role of interleukin-7 in degenerative and inflammatory joint diseases. *Arthritis Research & Therapy*.

[74] Silvestri T., Pulsatelli L., Dolzani P., Facchini A., Meliconi R. (2006). Elevated serum levels of soluble interleukin-4 receptor in osteoarthritis. *Osteoarthritis and Cartilage*.

[75] Yuan G. H., Masuko-Hongo K., Sakata M. (2001). The role of C-C chemokines and their receptors in osteoarthritis. *Arthritis and Rheumatism*.

[76] Scanzello C. R. (2017). Chemokines and inflammation in osteoarthritis: insights from patients and animal models. *Journal of Orthopaedic Research*.

[77] Vergunst C. E., van de Sande M. G., Lebre M. C., Tak P. P. (2009). The role of chemokines in rheumatoid arthritis and osteoarthritis. *Scandinavian Journal of Rheumatology*.

[78] Wojdasiewicz P., Poniatowski Ł. A., Szukiewicz D. (2014). The role of inflammatory and anti-inflammatory cytokines in the pathogenesis of osteoarthritis. *Mediators of Inflammation*.

[79] Robinson N. E., Robinson A. B. (2001). Deamidation of human proteins. *Proceedings of the National Academy of Sciences*.

[80] Catterall J. B., Hsueh M. F., Stabler T. V. (2012). Protein modification by deamidation indicates variations in joint extracellular matrix turnover. *Journal of Biological Chemistry*.

[81] Catterall J., Hsueh M. F., Stabler T. V., Renner J. M., Jordan J. M., Kraus V. B. (2011). 142 a unique deamidated cartilage oligomeric matrix protein (comp) biomarker preferentially identifies hip osteoarthritis. *Osteoarthritis and Cartilage*.

[82] Verzijl N., DeGroot J., Zaken C. B. (2002). Crosslinking by advanced glycation end products increases the stiffness of the collagen network in human articular cartilage: a possible mechanism through which age is a risk factor for osteoarthritis. *Arthritis and Rheumatism*.

[83] Šenolt L., Braun M., Olejárová M., Forejtová Š., Gatterova J., Pavelka K. (2005). Increased pentosidine, an advanced glycation end product, in serum and synovial fluid from patients with knee osteoarthritis and its relation with cartilage oligomeric matrix protein. *Annals of the Rheumatic Diseases*.

[84] Vos P. A. J. M., Mastbergen S. C., Huisman A. M. (2012). In end stage osteoarthritis, cartilage tissue pentosidine levels are inversely related to parameters of cartilage damage. *Osteoarthritis and Cartilage*.

[85] Zhang W., Randell E. W., Sun G. (2017). Hyperglycemia-related advanced glycation end-products is associated with the altered phosphatidylcholine metabolism in osteoarthritis patients with diabetes. *PLoS One*.

[86] Thijssen E., Van Caam A., Van Der Kraan P. M. (2015). Obesity and osteoarthritis, more than just wear and tear: pivotal roles for inflamed adipose tissue and dyslipidaemia in obesity-induced osteoarthritis. *Rheumatology*.

[87] Yang C. Y., Chanalaris A., Troeberg L. (2017). ADAMTS and ADAM metalloproteinases in osteoarthritis - looking beyond the 'usual suspects'. *Osteoarthritis and Cartilage*.

[88] Wachsmuth L., Bau B., Fan Z., Pecht A., Gerwin N., Aigner T. (2004). ADAMTS-1, a gene product of articular chondrocytes in vivo and in vitro, is downregulated by interleukin 1beta. *The Journal of Rheumatology*.

[89] Bekhouche M., Colige A. (2015). The procollagen N-proteinases ADAMTS2, 3 and 14 in pathophysiology. *Matrix Biology*.

[90] Yaykasli K. O., Hatipoglu O. F., Yaykasli E. (2015). Leptin induces ADAMTS-4, ADAMTS-5, and ADAMTS-9 genes expression by mitogen-activated protein kinases and NF-ĸB signaling pathways in human chondrocytes. *Cell Biology International*.

[91] Liu C. J., Kong W., Xu K. (2006). ADAMTS-12 associates with and degrades cartilage oligomeric matrix protein. *The Journal of Biological Chemistry*.

[92] Demircan K., Hirohata S., Nishida K. (2005). ADAMTS-9 is synergistically induced by interleukin-1*β* and tumor necrosis factor *α* in OUMS-27 chondrosarcoma cells and in human chondrocytes. *Arthritis and Rheumatism*.

[93] Luan Y., Kong L., Howell D. R. (2008). Inhibition of ADAMTS-7 and ADAMTS-12 degradation of cartilage oligomeric matrix protein by alpha-2-macroglobulin. *Osteoarthritis and Cartilage*.

[94] Conde J., Scotece M., Gomez R., Lopez V., Gomez-Reino J. J., Gualillo O. (2011). Adipokines and osteoarthritis: novel molecules involved in the pathogenesis and progression of disease. *Arthritis*.

[95] de Boer T. N., van Spil W. E., Huisman A. M. (2012). Serum adipokines in osteoarthritis; comparison with controls and relationship with local parameters of synovial inflammation and cartilage damage. *Osteoarthritis and Cartilage*.

[96] Zhang P., Zhong Z. H., Yu H. T., Liu B. (2015). Significance of increased leptin expression in osteoarthritis patients. *PLoS One*.

[97] Martín-Romero C., Santos-Alvarez J., Goberna R., Sánchez-Margalet V. (2000). Human leptin enhances activation and proliferation of human circulating T lymphocytes. *Cellular Immunology*.

[98] Koskinen A., Vuolteenaho K., Nieminen R., Moilanen T., Moilanen E. (2011). Leptin enhances MMP-1, MMP-3 and MMP-13 production in human osteoarthritic cartilage and correlates with MMP-1 and MMP-3 in synovial fluid from OA patients. *Clinical and Experimental Rheumatology*.

[99] Gómez R., Scotece M., Conde J., Gómez-Reino J. J., Lago F., Gualillo O. (2011). Adiponectin and leptin increase IL-8 production in human chondrocytes. *Annals of the Rheumatic Diseases*.

[100] Kroon F. P., Veenbrink A. I., de Mutsert R. (2019). The role of leptin and adiponectin as mediators in the relationship between adiposity and hand and knee osteoarthritis. *Osteoarthritis and Cartilage*.

[101] Figenschau Y., Knutsen G., Shahazeydi S., Johansen O., Sveinbjörnsson B. (2001). Human articular chondrocytes express functional leptin receptors. *Biochemical and Biophysical Research Communications*.

[102] Griffin T. M., Huebner J. L., Kraus V. B., Guilak F. (2009). Extreme obesity due to impaired leptin signaling in mice does not cause knee osteoarthritis. *Arthritis & Rheumatism: Official Journal of the American College of Rheumatology*.

[103] Fioravanti A., Cheleschi S., De Palma A. (2018). Can adipokines serum levels be used as biomarkers of hand osteoarthritis?. *Biomarkers*.

[104] Cheleschi S., Giordano N., Volpi N. (2018). A complex relationship between visfatin and resistin and microRNA: an in vitro study on human chondrocyte cultures. *International Journal of Molecular Sciences*.

[105] Scotece M., Koskinen-Kolasa A., Pemmari A. (2020). Novel adipokine associated with OA: retinol binding protein 4 (RBP4) is produced by cartilage and is correlated with MMPs in osteoarthritis patients. *Inflammation Research*.

[106] Yusuf E., Ioan-Facsinay A., Bijsterbosch J. (2011). Association between leptin, adiponectin and resistin and long-term progression of hand osteoarthritis. *Annals of the Rheumatic Diseases*.

[107] Kisluk J., Ciborowski M., Niemira M., Kretowski A., Niklinski J. (2014). Proteomics biomarkers for non-small cell lung cancer. *Journal of Pharmaceutical and Biomedical Analysis*.

[108] Alharbi R. A. (2020). Proteomics approach and techniques in identification of reliable biomarkers for diseases. *Saudi Journal of Biological Sciences*.

[109] Kong M. K., Min B. H., Lee P. C. (2012). Evaluation of a pretreatment method for two-dimensional gel electrophoresis of synovial fluid using cartilage oligomeric matrix protein as a marker. *Journal of Microbiology and Biotechnology*.

[110] Fernández-Costa C., Calamia V., Fernández-Puente P., Capelo-Martínez J. L., Ruiz-Romero C., Blanco F. J. (2012). Sequential depletion of human serum for the search of osteoarthritis biomarkers. *Proteome Science*.

[111] Hulme C. H., Wilson E. L., Fuller H. R. (2018). Two independent proteomic approaches provide a comprehensive analysis of the synovial fluid proteome response to autologous chondrocyte implantation. *Arthritis Research & Therapy*.

[112] Peffers M. J., Smagul A., Anderson J. R. (2019). Proteomic analysis of synovial fluid: current and potential uses to improve clinical outcomes. *Expert Review of Proteomics*.

[113] Mateos J., Lourido L., Fernández-Puente P. (2012). Differential protein profiling of synovial fluid from rheumatoid arthritis and osteoarthritis patients using LC-MALDI TOF/TOF. *Journal of Proteomics*.

[114] Hsueh M. F., Önnerfjord P., Kraus V. B. (2014). Biomarkers and proteomic analysis of osteoarthritis. *Matrix Biology*.

[115] Kriegsmann M., Seeley E. H., Schwarting A. (2012). MALDI MS imaging as a powerful tool for investigating synovial tissue. *Scandinavian Journal of Rheumatology*.

[116] Zhen E. Y., Brittain I. J., Laska D. A. (2008). Characterization of metalloprotease cleavage products of human articular cartilage. *Arthritis & Rheumatism: Official Journal of the American College of Rheumatology*.

[117] Han M. Y., Dai J. J., Zhang Y. (2012). Identification of osteoarthritis biomarkers by proteomic analysis of synovial fluid. *Journal of International Medical Research*.

[118] Ritter S. Y., Subbaiah R., Bebek G. (2013). Proteomic analysis of synovial fluid from the osteoarthritic knee: comparison with transcriptome analyses of joint tissues. *Arthritis and Rheumatism*.

[119] Zhu Z., Zhong L., Li R. (2020). Study of osteoarthritis-related hub genes based on bioinformatics analysis. *BioMed Research International*.

[120] Wang X., Ning Y., Guo X. (2015). Integrative meta-analysis of differentially expressed genes in osteoarthritis using microarray technology. *Molecular Medicine Reports*.

[121] Hu Z. Z., Huang H., Wu C. H. (2011). Omics-based molecular target and biomarker identification. *Bioinformatics for Omics data*.

[122] Tachmazidou I., Hatzikotoulas K., Southam L. (2019). Identification of new therapeutic targets for osteoarthritis through genome- wide analyses of UK Biobank data. *Nature Genetics*.

[123] Brooks J., Watson A., Korcsmaros T. (2017). Omics approaches to identify potential biomarkers of inflammatory diseases in the focal adhesion complex. *Genomics, Proteomics & Bioinformatics*.

[124] Zhu N., Hou J., Wu Y. (2018). Identification of key genes in rheumatoid arthritis and osteoarthritis based on bioinformatics analysis. *Medicine*.

[125] Zhai G., Randell E. W., Rahman P. (2018). Metabolomics of osteoarthritis: emerging novel markers and their potential clinical utility. *Rheumatology*.

[126] Zhang W., Sun G., Likhodii S. (2016). Metabolomic analysis of human plasma reveals that arginine is depleted in knee osteoarthritis patients. *Osteoarthritis and Cartilage*.

[127] Yang G., Zhang H., Chen T. (2016). Metabolic analysis of osteoarthritis subchondral bone based on UPLC/Q-TOF-MS. *Analytical and Bioanalytical Chemistry*.

